# DES-Tcell is a knowledgebase for exploring immunology-related literature

**DOI:** 10.1038/s41598-021-93809-1

**Published:** 2021-07-12

**Authors:** Ahdab AlSaieedi, Adil Salhi, Faroug Tifratene, Arwa Bin Raies, Arnaud Hungler, Mahmut Uludag, Christophe Van Neste, Vladimir B. Bajic, Takashi Gojobori, Magbubah Essack

**Affiliations:** 1 Department of Medical Laboratory Technology (MLT), Faculty of Applied Medical Sciences (FAMS), King Abdulaziz University (KAU), Jeddah, 21589-80324 Saudi Arabia; 2 Computer, Electrical, and Mathematical Sciences and Engineering Division (CEMSE), Computational Bioscience Research Center (CBRC), King Abdullah University of Science and Technology (KAUST), Thuwal, 23955-6900 Saudi Arabia

**Keywords:** Immunological disorders, Immunology, Data mining

## Abstract

T-cells are a subtype of white blood cells circulating throughout the body, searching for infected and abnormal cells. They have multifaceted functions that include scanning for and directly killing cells infected with intracellular pathogens, eradicating abnormal cells, orchestrating immune response by activating and helping other immune cells, memorizing encountered pathogens, and providing long-lasting protection upon recurrent infections. However, T-cells are also involved in immune responses that result in organ transplant rejection, autoimmune diseases, and some allergic diseases. To support T-cell research, we developed the DES-Tcell knowledgebase (KB). This KB incorporates text- and data-mined information that can expedite retrieval and exploration of T-cell relevant information from the large volume of published T-cell-related research. This KB enables exploration of data through concepts from 15 topic-specific dictionaries, including immunology-related genes, mutations, pathogens, and pathways. We developed three case studies using DES-Tcell, one of which validates effective retrieval of known associations by DES-Tcell. The second and third case studies focuses on concepts that are common to Grave’s disease (GD) and Hashimoto’s thyroiditis (HT). Several reports have shown that up to 20% of GD patients treated with antithyroid medication develop HT, thus suggesting a possible conversion or shift from GD to HT disease. DES-Tcell found miR-4442 links to both GD and HT, and that miR-4442 possibly targets the autoimmune disease risk factor CD6, which provides potential new knowledge derived through the use of DES-Tcell. According to our understanding, DES-Tcell is the first KB dedicated to exploring T-cell-relevant information via literature-mining, data-mining, and topic-specific dictionaries.

## Introduction

### The role of T-cells in the immune system

The immune system is a complex network of cells, tissues, and molecules working together to defend the body against invading organisms, and abnormal cancerous cells. It is composed of two cooperative arms including innate (or nonspecific) immunity, and adaptive (or specific) immunity^1,2^. One of the critical features of the immune system is self-discrimination, which refers to its ability to distinguish self (the body) from non-self (foreign invaders)^3,4^. Lymphocytes, the major cellular component of the immune system, are classified into two main types: T lymphocytes (T-cells) and B lymphocytes (B-cells). Both B- and T-cells originate from pluripotent hematopoietic stem cells in the bone marrow. However, only B-cells mature there, while T-cells migrate to the thymus where they undergo a maturation process. When these newly generated naive cells undergo maturation and activation, they proliferate and differentiate into effector and memory subtype cells that have distinct functions. T-cells are specialized immune cells of the adaptive immune system and key players in mediating cellular immunity against abnormal cells, including pathogen-infected cells and cancer cells. They express a unique surface receptor called T-cell receptor (TCR) that recognizes a specific antigenic determinant displayed by a major histocompatibility complex (MHC) molecules on the surface of other cells, such as antigen-presenting cells (APCs)^4^. The majority of T-cells express αβ TCR and either CD4 or CD8 molecules (also known as αβ T-cells), whereas a small proportion of “unconventional” T-cells express γδ TCR (also known as γδ T-cells)^5,6^. Different types of T-cells perform various functions including: (a) CD8 + cytotoxic T-cells recognize intracellular antigens and directly kill cells infected with intracellular pathogens (e.g., viral-infected cells) and tumor cells; (b) CD4 + helper T-cells recognize extracellular antigens and indirectly help with the clearing of extracellular pathogens by secreting cytokines that can enhance phagocytic cells (e.g., macrophages) and other T-cells; (c) regulatory T-cells are suppressor cells that play an essential role in regulating immune responses as well as maintaining immunological tolerance by preventing autoreactive T-cell activation in the periphery. Also, there are other subsets of T-cells include gamma delta T-cells (γδ T-cells), natural killer T-cell (NKT) and mucosal-associated invariant T-cells (MAIT)^7–9^.

Although T-cells play a crucial role in protective immunity, a hindrance of their functioning can cause immune-related diseases. For example, polymorphisms in CTLA-4 gene, which encodes a protein that delivers an inhibitory signal to T-cells, have been associated with autoimmune diseases, such as Grave’s disease, systemic lupus, and type 1 diabetes^10,11^. Moreover, genetic engineering of T-cells offers new therapeutic potential for cancer, infectious, and autoimmune diseases. For example, the adoptive transfer of genetically engineered T-cells redirected toward a specific tumor antigen has shown promising results in treating cancer, especially hematological malignancies^12^. However, many factors limit T-cell therapy, such as finding a potential target antigen and identifying new potential stimulatory/inhibitory molecules that can enhance the therapeutic T-cell efficacy and safety^13^.

These findings, amongst others, maintain interest in T-cell-related research. In fact, querying Web of Science (All Databases) in Clarivate Analytics (https://clarivate.com/) on 26 July 2019 using the query [T-cell* OR “T cell” OR “T cells” OR “T lymphocyte” OR “T lymphocytes” OR “T-lymphocyte” OR “T-lymphocytes” OR antigen* OR antibody OR autoimmun*], to retrieve all T-cell-related research, produced 509,627 articles published in the last five years. This number of published articles indicates that on average, ~ 102,000 articles are published yearly, a volume of information that would be challenging to analyze.

### Exploring the plethora of published information

Given the enormous volume of scientific literature, it is necessary to deploy automated tools and resources for information search, retrieval, analysis, summarization, and hypotheses generation to expand domain-specific knowledge. In this regard, natural language processing (NLP), text-mining, and information integration systems are being used to tackle different biomedicine and life sciences problems. For example, studies demonstrate using text-mining to extract genotype–phenotype relationships^14^, identify patients with trileaflet aortic stenosis and coronary artery diseases^15^, extract terminologies of sleep disorders from journal articles^16^, and mining adverse drug events^17^. Moreover, applying text-mining in cancer research^18^, and psychiatry studies^19^. Processing scientific literature using text-mining approaches has also become a common theme^20,21^. Examples of such systems include:

- Cancer Hallmark Analytics Tool (CHAT): for organizing and analyzing cancer-related literature^22^
- DISEASES is a text-mining and data integration system of associations between genes and diseases^23^
- PolySearch2: for discovering associations between multiple entities, including human diseases, genes, drugs, metabolites, and toxins^24^
- DES-RedoxVasc: for summarizing the literature on redox control of vascular biology^27^
- DDMGD: for extracting associations between methylated genes and diseases from scientific literature^28,29^.

The extracted information is saved in knowledgebases (KBs), facilitating storing, searching, retrieving, and visualizing scientific information. The biomedical domain has benefitted from the development of several of these KBs. For example, Mouse Genome Informatics (MGI) is a resource for genetic, genomic, and biological information related to the laboratory mouse^30^. Norine is a KB dedicated to providing information related to non-ribosomal peptides^31^. Also, DMAK is a curated KB for pan-cancer DNA methylation^32^. Other cancer-related KBs are CanSar for cancer research and drug discovery^33^, the literature-based ECGene KB for endometrial cancer genes^34^, and PCOSKB associated with PolyCystic Ovary Syndrome^35^ that provide concepts from genes, diseases, ontology terms, and biochemical pathways. There is also SwissLipids, a KB for lipid biology^36^, and several other KBs developed for other domains^37–43^. Here we developed DES-Tcell for the exploration of immunology-related information, with a particular focus on T-cells.

## The DES-Tcell exploration system

We created the DES-Tcell KB on June 20, 2020. DES-Tcell is developed based on the Dragon Exploration System (DES), used as the underlying framework for several topic-specific KBs^25,26,28,29,45–58^. The DES workflow has been described earlier^44^. In brief, we created a T-cell-specific literature corpus, as well as compiled topic-specific dictionaries, which are used to extract term-document mappings, subsequently used to determine statistically enriched concepts and pairs of concepts.

### Literature corpus

To create the literature corpus incorporated into DES-Tcell, we queried the PubMed and PubMed Central articles in our local MongoDB repository for all T-cell-related articles. The query [T-cell* OR “T cell” OR “T cells” OR “T lymphocyte” OR “T lymphocytes” OR “T-lymphocyte” OR “T-lymphocytes” OR antigen* OR antibody OR autoimmun*] was used to retrieve the T-cell-related articles from PubMed and PubMed Central resources, which serve as the literature corpus. To ensure extraction of all the T-cell-related articles, the matching system used finds all documents with terms from the query. In this case, the literature corpus comprises more than 1.4 million articles.

### Topic-relevant dictionaries

There are 15 topic-relevant dictionaries incorporated into this KB, which include the newly compiled dictionary, “Immunology Related (Various)” (see Table 1). The other 14 dictionaries were compiled and used in previously published KBs, developed through the DES framework (see Table 1). Thus, in Table 1, these 15 dictionaries are referred to as “pre-existing in DES.”

**Table 1:** List of dictionaries used in DES-Tcell with source references.

Dictionaries	Enriched unique terms in the KB	Source
**Chemicals/compounds**		
Chemical Entities of Biological Interest (ChEBI)	12,107	Pre-existing in DES
**Functional annotation**		
Biological process (GO)^59^	4439	pre-existing in DES
Cellular component (GO)^59^	1270	pre-existing in DES
Molecular function (GO)^59^	1213	pre-existing in DES
Pathways (KEGG^60^, reactome^61^, unipathway^62^, PANTHER^63^)	1079	pre-existing in DES
**Diseases**		
DOID ontology (bioportal) human disease ontology^64^	4290	pre-existing in DES
HP ontology (bioportal) human phenotype ontology^65^	3611	pre-existing in DES
SIDER (drug indications and side effects)^66^	3518	pre-existing in DES
**Drugs**		
Drugs (DrugBank)^67^	3319	pre-existing in DES
**Human**		
Human genes and proteins (EntrezGene)^68^	19,654	Pre-existing in DES
Human long non-coding RNAs^69^	228	Pre-existing in DES
Human microRNAs (HGNC^69^ and Entrez gene^68^)	1530	Pre-existing in DES
Mutations (tmVar)^70^	15,925	Pre-existing in DES
**Immunology**		
Immunology related (various)	1913	Newly compiled
**Anatomies**		
Human anatomy (in-house compiled)	2634	Pre-existing in DES

All dictionary concepts are normalized to ensure that a concept referred to in the text by different symbols, names or synonyms is recognized as a single entity. For example, routinely used names/symbols/aliases in trusted sources such as Entrez Gene (for genes) with UniProt (for proteins) nomenclatures, are combined, to represent the genes and proteins they translate into as a single entity applied in the DES system. This normalization improves the accuracy of concepts’ enrichment estimates.

### Concept-document mapping

We used the literature corpus and 15 dictionaries for concept-document mapping. This mapping information was used to determine the concepts most relevant to the T-cell functions. A concept was determined to be relevant to the KB if the frequency of the concept in the KBs literature corpus is significantly higher than its frequency in the full set of articles or background set. Specifically, we identified a concept as an enriched concept, if the concept had a false discovery rate (FDR) < 0.05 in the DES-Tcell literature corpus when compared to the complete set of PubMed and PubMed Central articles in our local MongoDB literature repository. FDR was calculated based on the Benjamini–Hochberg procedure to correct for multiplicity testing.

We also determined enriched pairs of concepts, based on the abundance of concepts co-occurrence as compared to the occurrence of the individual concepts. These pairs of concepts may or may not be associated, but there is a high probability that the concept pairs are associated when concept pairs are enriched.

From all the concepts in the 15 dictionaries used in DES-Tcell, 76,748 concepts were determined as statistically enriched concepts, and 7,230,602 concept pairs were determined as statistically enriched pairs of concepts (see KB statistics in Table 2). The number of enriched concepts per dictionary is provided in Table 1.

**Table 2:** Statistics of the DES-Tcell knowledgebase.

Statistics	Value
Number of significantly enriched concepts	76,748
Number of significantly enriched pairs of concepts	7,230,602
Total number of articles included in DES-Tcell	1,425,007
Number of articles that contain the term “T cell proliferation”	13,762
Number of concepts associated with the concept “abnormal T cell activation” using semantic similarity	137,377

We also computed the semantic similarity between any two concepts within the KB. The similarity metric indicates that there is a likeness or closeness between two concepts, in DES-Tcell. Likeness or closeness based on concept co-occurrence may not be direct. The cosine distance between two concept embeddings was used to calculate semantic similarity in DES-Tcell. We obtained these embeddings from a skip-gram Word2Vec model trained on the DES-Tcell literature corpus. The literature corpus, the 15 dictionaries, the enriched concepts, the enriched pairs of concepts, and semantically related concepts were integrated to create DES-Tcell.

## Knowledgebase utilities and case studies

We designed DES-Tcell to allow easy exploration of concepts enriched in immunology-related literature focused on T-cells. Salhi et al., provide a detailed description of several links provided in DES-Tcell that facilitates the exploration of concepts in different contexts^55^. Briefly, users can explore the enriched concepts in immunology-related information via the “Enriched Concepts” link. One can also explore the enriched co-occurring concepts (in the title/abstract level and full-text document when the concepts are within 200-character proximity) using the “Enriched Pairs” link. To further facilitate the natural exploration of literature, users can also use the “Column visibility” tab. This tab provides links to explore enriched concepts using ranking options for false discovery rate (FDR), density, term frequency within KB, and term frequency in literature. FDR is calculated based on the Benjamini–Hochberg algorithm. Also, all concepts are in topic-specific dictionaries that are color-coded, and each concept can be clicked on to explore its “Network” and “Term Co-occurrence” links. The “Network” link allows users to generate multilayered networks of associated biomedical concepts. Also, a “Literature” link allows users to explore the literature associated with the enriched concepts in DES-Tcell. Furthermore, DES also provides a new “Semantic Similarity” link described in Essack et al.^27^. Here, it should be recognized that links found through semantic similarity is not as obvious as links found simply through co-occurrence of concepts or concept being linked through a common factor as semantic similarity offers concept associations based on a Word2Vec embedding of the corpus, as described above.

*Case study 1*: Using “Enriched Concept” to demonstrate that DES-Tcell retrieves reasonable associations.

**Figure 1:**
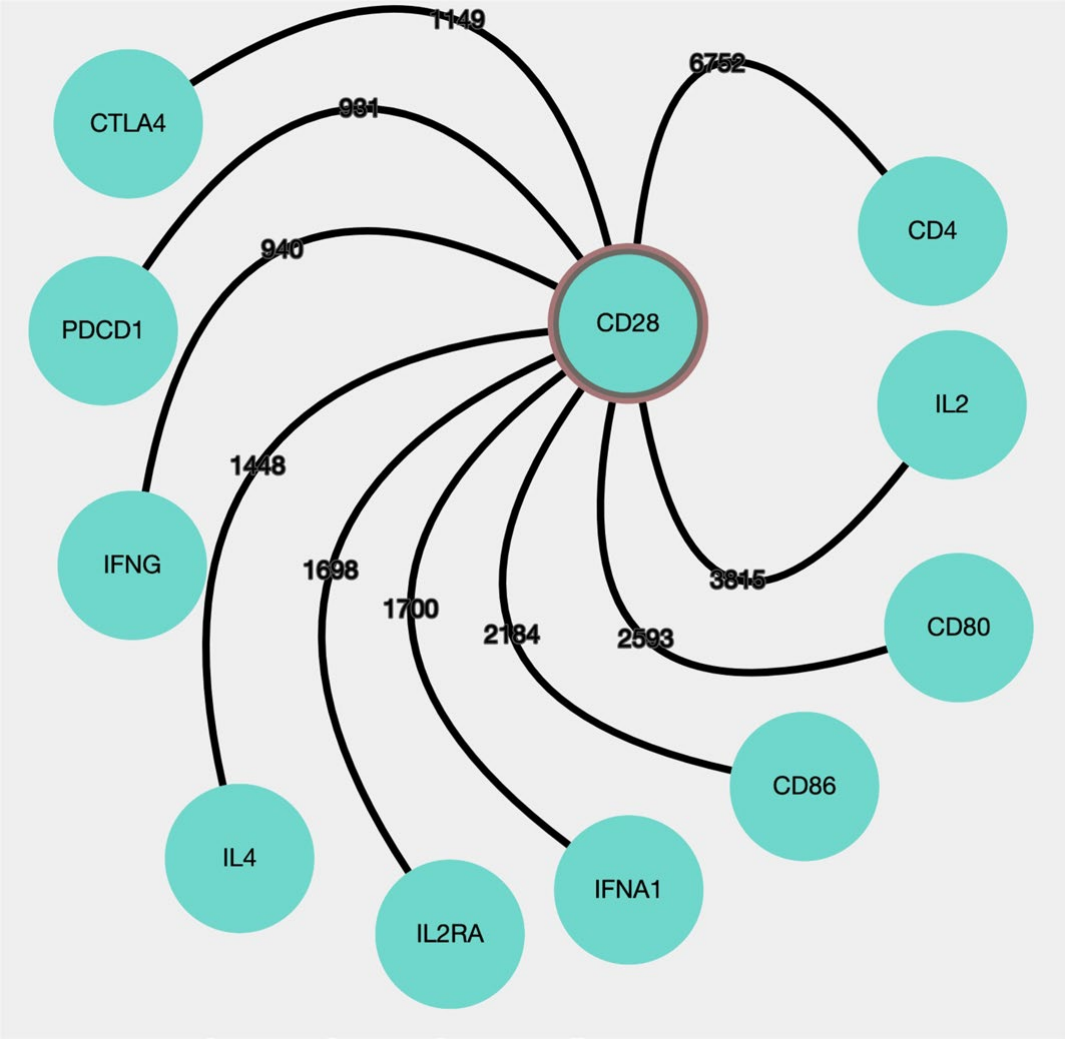
Illustrates the efficacy of DES-Tcell to retrieve established or strong associations. Only the top 10 associations of genes/proteins linked to CD28 as found in the DES-Tcell KB are shown. The nodes represent concepts from the “immunology related (various)” dictionary. Depending on the co-occurrence frequency, the color of edges will range from black (strong association) to grey (weaker association), and the number on each edge represents the number of documents that link the potentially associated nodes.

DES-Tcell can be used to quickly develop an insight to the concepts that are most relevant in immunology-related to T-cells. For example, to find out which immunology-related genes are involved in T-cell activation, the user may choose the “Enriched Pairs” view button (in the left side panel). This page will display two columns; in the first column insert “regulation of T-cell activation” and select the “Immunology Related (Various)” in the second column (sorted by FDR). The list of immunology-related genes, most often associated with T-cell activation in the text, will be displayed. In the list, we selected the concept “CD28”, then used this concept right-click menu to generate a network (Fig. 1, Step 1). On the “Network” page, we selected the “Immunology Related (Various)” dictionary. Then, the “CD28” node was highlighted and expanded by the associated terms from the selected dictionary (Fig. 1, Step 2).

To confirm the genes nodes are known or valid associations retrieved by DES-Tcell, we checked the literature suggested by DES-Tcell. Past research shows that all genes/proteins retrieved are associated with T-cell activation. That is, APCs fully activate the T-cell via receiving three signals that include TCR signaling which is triggered when the TCR together with the CD4/CD8 co-receptors recognize a peptide/MHC complex (signal1), in combination with co-stimulatory signal (signal 2)^71^. CD28 and cytotoxic T-lymphocyte antigen-4 (CTLA-4) are proteins/receptors on the T-cell surface that provide co-stimulatory or co-inhibitory signals, respectively, amongst others (such as ICOS and PD-1) which are involved in the regulation of T-cell activation by APCs^72^. Both CD28 and CTLA-4 bind to the same receptor on APC called B7 molecules (CD80/CD86)^73^, which are upregulated upon activation by the innate immune responses, but the outcome will be different, that is: 1/ engagement of CD28 will result in T-cell activation, and 2/engagement of CTLA-4 will inhibit the T-cell activation (act as a suppressor). Following stimulation of T-cells by TCR/CD28 signaling, the activated T-cells in their clonal expansion and differentiation into effector cells secrets IL-2 cytokine. This stimulation can also be enhanced by a third signal that is provided by pro-inflammatory cytokines produced by APCs (macrophages and/or dendritic cells), cytokines such as IL-2, IFNγ, and IL-12^74,75^. High-affinity IL-2 receptor (IL2R) is also induced upon T-cell activation resulting in the enhanced T-cell ability of binding and responding to secreted IL-2, an essential cytokine in enhancing T-cell proliferation and survival^76^. Taken together, our literature search shows that all genes/proteins nodes in Fig. 1 have a valid association with CD28.

Despite these true associations, during the literature search process, we observed several limitations. For example, some genes/proteins synonyms are ambiguous with general terms, such as CAN, which results in false-positive associations. Also, normalizing the terms/phrases does not entirely eradicate inconsistencies in disease nomenclature. Furthermore, the linked nodes in the generated networks do not necessarily reflect a direct relationship but rather indicate two concepts discussed in the same text.

*Case study 2*: miR-4442 possibly targets CD6 and suggests novel insight derived through the use of DES-Tcell.

Grave’s disease is an autoimmune disorder characterized by the production of autoantibodies against TSHR that causes hyperthyroidism^77,78^. On the other hand, Hashimoto’s thyroiditis (HT) is an autoimmune disorder that is characterized by the production of autoantibodies against thyroid peroxidase (TPO) that causes hypothyroidism^79^. However, autoantibodies against TPO were also found present in GD patients^80^, suggesting a close association between these diseases. Several reports have shown that up to 20% of GD patients treated with antithyroid medication develop HT^81–84^. Thus, there is a possibility of conversion or shift from GD to HT disease. There is also a role of Treg in the natural progression of GD to HT. Moreover, there is a link between these diseases and mutations in CTLA-4 and CD28 genes that are associated with T-cell activation. New insights could help to increase our understanding of the underlying mechanisms responsible for the shift between diseases.

**Figure 2:**
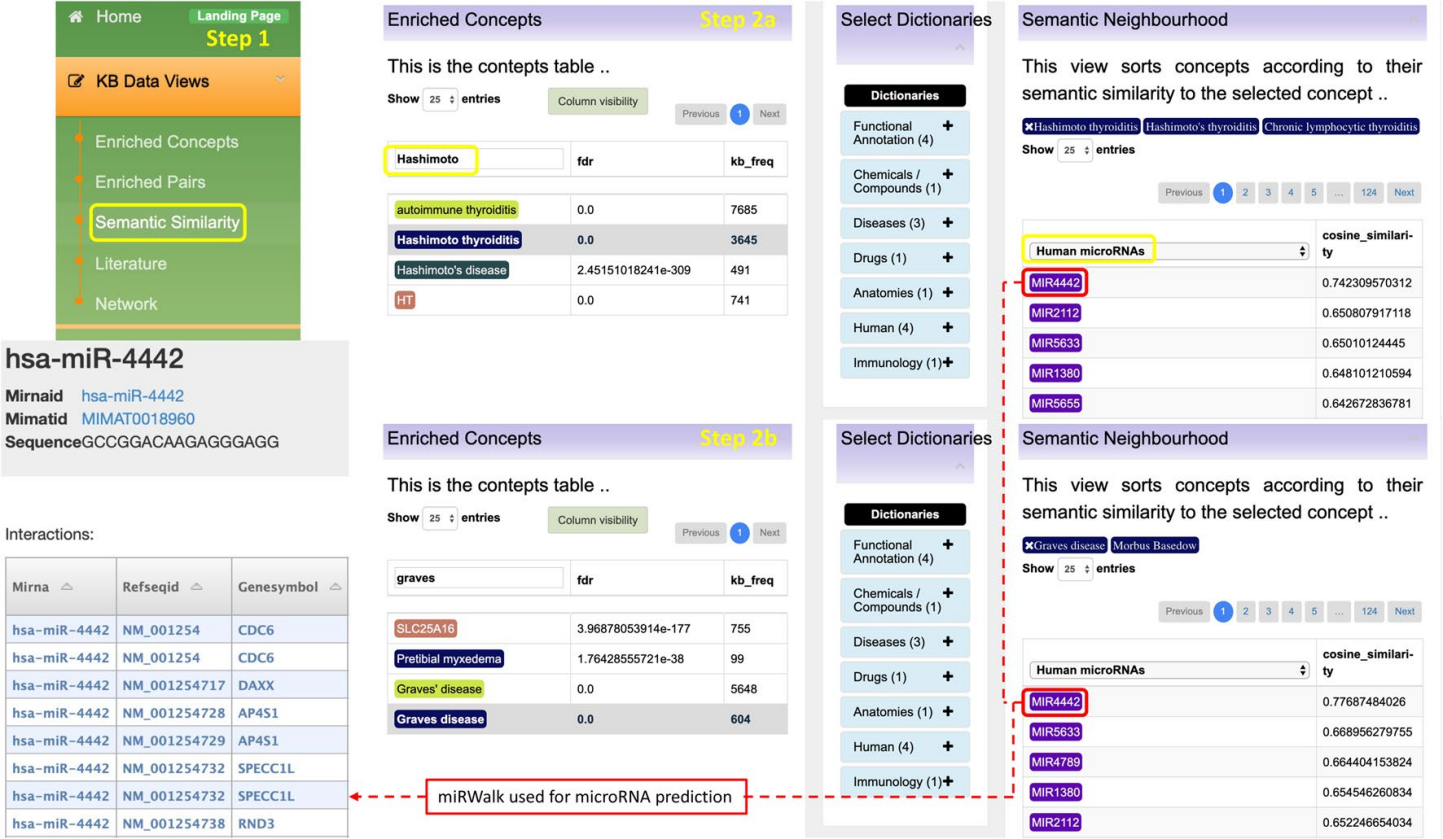
The step-by-step illustration of how DES-Tcell can be used to identify microRNA’s potentially association to Grave’s disease (step 2a) and Hashimoto’s thyroiditis (step 2b) and provide the potential target of the identified microRNA.

In search of novel insights, we also looked at the associations of concepts based on semantic similarity using the “Semantic Similarity” link. In the search bar, we typed the concept of interest “Hashimoto”, then select the “Human microRNAs” dictionary to view the concepts in this dictionary that have semantic similarity to our selected concept “Hashimoto” (Fig. 2, step2a). This process was repeated using another concept, “Graves” (Fig. 2, step2b). We find four of the five top-ranked concepts, “MIR4442”, “MIR2112”, “MIR5633”, and “MIR1380” being linked to both GD and HT. However, current published literature could not provide any information about these miRNAs’ functions in GD or HT. Consequently, we used miRWalk for miRNA target prediction^85^. We did not retrieve any result for “MIR2112”, “MIR5633”, and “MIR1380 but retrieved several predicted targets of “MIR4442” including CD6. CD6 is a cell surface marker expressed on immature thymocytes, the majority of mature T-cells and a subset of B-cells and NK cells. Its expression is induced by CD2 upon thymocyte maturation and following T-cell activation, and CD6 binds to its broadly expressed ligand CD166/ALCAM (activated leukocyte cell adhesion molecule)^86^. Thus, it promotes T-cell adhesion to APCs and involves in the immunological synapses’ stabilization and maturation^87^. In addition, CD6 synergizes with TCR or CD28 as a co-stimulatory molecule that can enhance T-cell activation and proliferation^87–90^. Singer et al. has shown that CD6 is also involved in the survival and selection of thymocyte in humans and mice^89^. Recently, CD6 is found to be a risk factor for some autoimmune diseases such as multiple sclerosis and Behcet’s disease^91,92^. Accordingly, anti-CD6 monoclonal antibodies have been developed as a potential therapy for autoimmune diseases, which have been shown to restrain the proliferation of autoreactive T-cells or antigen-dependent activation of T-cells^93^. As CD6 expression is also increased in autoreactive T-cells, studies showed that monoclonal antibody targeting CD6 (UMCD6) could inhibit a clone of autoreactive T-cells in vitro^94^. Taken together, although current literature shows no evidence for microRNA “MIR4442” functions in GD or HT diseases, DES-Tcell suggests potential association of this microRNA to GD/HT diseases and the possibility of involvement in GD conversion to HT. Therefore, DES-Tcell associations are suggested as potentially new immunological knowledge.

*Case study 3*: DES-Tcell suggests MEG3 may be a potential therapeutic target for GD.

**Figure 3:**
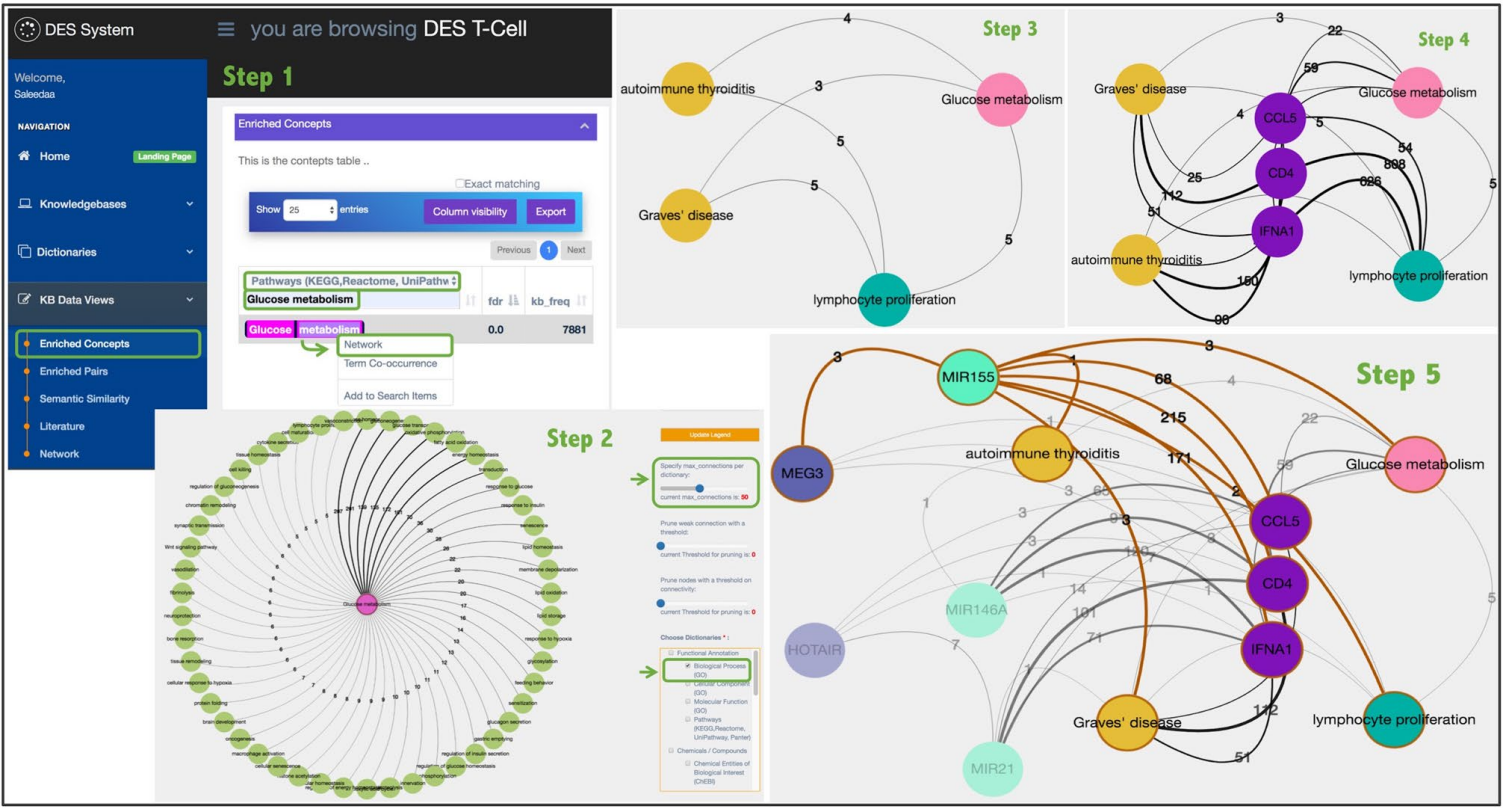
Step-by-step illustration of how DES-Tcell can be used to explore potentially new therapeutic targets for Graves’ disease.

Alteration in the metabolic activity is required for T-cell proliferation, differentiation, and effector functions^95^. That is, metabolism is increased upon T-cell activation, and it has been demonstrated that glucose deprivation resulted in impaired lymphocyte activation and function^96^. Thus, we further explore this association between glucose metabolism and lymphocyte proliferation using “Enriched Concepts”. We started this process by filtering the “Pathways (KEGG, Reactome, Unipathway, Panter)” dictionary with ‘Glucose metabolism’ and then generated a network from the right-click menu attached to the “Glucose metabolism” concept (Fig. 3, step 1). To find the concept associated with this node, we then selected the “Biological process (GO)” dictionary, specified 50 nodes as the maximum connection, and highlighted the “Glucose metabolism” node to select “Expand from this term” (Fig. 3, step 2). This process retrieved several biological processes, but we removed all the nodes except “lymphocyte proliferation” to ensure that the retrieved nodes in subsequent steps are linked to these two concepts. We then selected the Disease Ontology “DOID Ontology (Bioportal)” dictionary and expanded the “Glucose metabolism” and the “lymphocyte proliferation” nodes with terms from these dictionaries. We also simplified the resulting network by removing all nodes with a single link. Here, we retrieved several concepts from the “DOID Ontology (Bioportal)” dictionary including different types of cancer (such as “T-cell leukemia”, “Burkitt lymphoma”, “thymoma”, “Kaposi’s sarcoma” and “colon carcinoma”), autoimmune diseases (such as “Graves’ disease”, “autoimmune thyroiditis”, and “type 1 diabetes mellitus”), infectious diseases (such as “salmonellosis”, “typhoid fever” and “visceral leishmaniasis”) and other syndromes/conditions (such as “malnutrition”, “chronic fatigue syndrome”, “transient myeloproliferative syndrome”, and “systemic inflammatory response syndrome”) were linked to both the “lymphocyte proliferation” and “glucose metabolism” nodes. However, since lymphocyte proliferation is induced by thyroid autoantigens in autoimmune thyroid diseases such as Graves’ disease and autoimmune thyroiditis^97^, we removed all disease nodes except “Graves’ disease” and “autoimmune thyroiditis” (also known as Hashimoto’s thyroiditis (HT)) (Fig. 3, step 3). Moreover, it has been shown that hyperthyroidism and hypothyroidism caused by GD and HT, respectively, can result in glucose intolerance and insulin resistance^98,99^. To expand the associations, we specified 20 nodes as the maximum connection, and we chose to expand from all nodes including the “Graves’ disease”, “autoimmune thyroiditis” “Glucose metabolism” and “lymphocyte proliferation” by clicking on “Immunology Related (Various)” dictionary only. Then, we simplified the resulting network by removing all nodes with two links (Fig. 3, step 4). This revealed a set of genes including “CD4”, “IFNA1” and “CCL5” that are linked to “lymphocyte proliferation” and “glucose metabolism” and also linked to both diseases including “Graves’ disease” and “autoimmune thyroiditis”. Expanding all three genes (“CD4”, “IFNA1” and “CCL5”) and other nodes in our network with the “Human microRNAs” dictionary and simplifying the resulted network by removing all nodes with 3 links showed that all the three genes are controlled by different miRNAs including miR-155, miR-146, and miR-21. As miRNAs’ functions can be controlled by long non-coding RNA (lncRNA), we chose to expand all miRNAs as well as all other nodes in our network with the “Human Long Non-Coding RNAs” dictionary, and once again, the resulted network was simplified by removing all nodes with three links. The resulting network revealed that all three genes are essentially controlled by ‘miR-155’ and that maternally expressed gene 3 ‘MEG3’ is the only lncRNA associated with ‘miR-155’ (Fig. 3, step 5).

‘CCL5’ is an essential proinflammatory chemokine (also called RANTES) that regulates cell metabolism in activated T cells (including CD4^+^ helper T cells) as well as cell migration (chemotaxis) to the site of inflammation via activation of its cognate receptor including CCR5^100,101^. CCL5-CCR5 signaling induces cellular metabolic activity by increasing the expression of glucose transporter 1 (Glut1), glucose uptake (mTOR-dependent), and intracellular ATP levels^101,102^. Increased levels of serum CCL5 have been observed in GD patients as well as impaired glucose tolerance (IGT), insulin resistance (IR) and insulin secretion^99,103,104^.

Another subset of CD4^+^ T cells, called regulatory T cells (Tregs), which plays an indispensable role in preserving immunological tolerance to self-antigens as well as homeostasis, is also involved in the development of GD^105^. Decreased numbers of Tregs and impairment in their immunosuppressive functions have been observed in GD patients^106^. ‘miR-155’ is highly expressed in Tregs and regulated by the transcription factor FOXP3 (forkhead box protein P3), which acts as a master regulator for Treg development and function^107,108^. Another miRNA that appeared in our network is ‘miR-146a’ which is ubiquitously expressed in Tregs and plays a pivotal role in Treg cell-mediated suppressive function^109^. Both ‘miR-155’ and ‘miR-146a’ levels of expression were found to be significantly low in GD patients compared to healthy controls^110^. Thus, decreased expression of ‘miR-155’ may positively correlate with decreased FOXP3 expression and Treg numbers and impaired Treg function was seen in GD patients^110,111^.

Taken together, although current literature shows no evidence for “MEG3” functions in GD, DES-Tcell suggests a potential association of this lncRNA to GD. This idea is strengthened by the Yu et al. study that shows ‘miR-155’ (that we know to be involved in GD) is a direct target of the ‘MEG3’ which acts by sponging ‘miR-155’ in acute myeloid leukemia (AML) cells^112^. Therefore, this DES-Tcell association is suggested as potentially new immunological knowledge and should be considered a potential therapeutic target.

## Concluding remarks

DES-Tcell allows for exploration of numerous concepts and associated pairs of concepts related to immunology, based on analyzed literature and associated data-mining. Topic-specific dictionaries provide the concepts used. DES-Tcell has identified over 7.2 million enriched associations of concepts, based on the co-occurrence of the concepts within 200 characters distance. These associated concepts are enriched, when compared to documents in the background (all articles in PubMed and PMC in our local repository), as well as the individual concepts they comprise of. We further provide an additional set of associated concepts based on semantic similarity between any of the enriched concepts and dictionary concepts. These associations are likely meaningful if they have > 0.75 similarities and DES-Tcell provides more than 10 million such associations.

The case studies demonstrate that this system can retrieve valid associations and suggest potentially new insights. However, the new insights indicated in the case studies are neither experimentally validated statements nor automatically generated hypotheses, but a proposition made by the authors by exploring the relevant links among the discussed entities, as detailed in the case study. Previous versions of DES included a hypothesis generator, but this was abandoned in favor of leaving this task to the user. The reason being, as the practical use of the system, showed that automatically generated hypotheses were seldom used, mainly due to 1/their sheer number (combinatorial explosion from the knowledge graph), even when ranking and stringent cut-offs are applied, hence, they inflate the KB unnecessarily, and 2/starting with a concept, or an association, and their context within the literature is the more natural way of exploring related information.

DES-Tcell carries all shortcomings similar to other text-mining-based approaches. The KB uses a dictionary-based system for indexing, which has been a widely used approach so far. However, with recent breakthroughs in NLP research, mainly thanks to novel deep-learning architectures and vast amounts of training data, jumps in performance in various NLP tasks such as text-generation and prediction spur applying such research to biomedical text-mining again. Better entity recognition models can mitigate dictionary cleaning, term ambiguity, and promiscuous terms-related problems introduced by controlled vocabularies. Such issues stem from the use of pre-defined terms rather than extracting mentions dependent on context. However, adopting such models would introduce major changes to the system, so it is within our long-term goals.

Nonetheless, for now, our system provides indexing of a comprehensive list of dictionaries relevant to immunology into the literature. Hence allowing quick access to term mentions in context.

- Term associations across documents.
- Potentially insights into missing links.

The user interface provides searching, filtering, and sorting functionality. The network tab provides a quick and accessible exploration of small knowledge-graphs, centered around several entities of interest at once. We believe the knowledge base would be an asset to the immunology research community, as it indexes more than 1 M documents with a wealth of biological entities. Also, we will update the KB biannually to include newly published articles and updated dictionaries.

## Data Availability

The DES-Tcell portal is free for academic and nonprofit users and can be accessed at http://cbrc.kaust.edu.sa/des-tcell/.
