# Author Correction: DES-Tcell is a knowledgebase for exploring immunology-related literature

**DOI:** 10.1038/s41598-021-01795-1

**Published:** 2021-11-10

**Authors:** Ahdab AlSaieedi, Adil Salhi, Faroug Tifratene, Arwa Bin Raies, Arnaud Hungler, Mahmut Uludag, Christophe Van Neste, Vladimir B. Bajic, Takashi Gojobori, Magbubah Essack

**Affiliations:** 1 Department of Medical Laboratory Technology (MLT), Faculty of Applied Medical Sciences (FAMS), King Abdulaziz University (KAU), Jeddah, 21589‑80324 Saudi Arabia; 2 Computer, Electrical, and Mathematical Sciences and Engineering Division (CEMSE), Computational Bioscience Research Center (CBRC), King Abdullah University of Science and Technology (KAUST), Thuwal, 23955‑6900 Saudi Arabia

Correction to: *Scientific Reports* 10.1038/s41598-021-93809-1, published online 12 July 2021

The original version of this Article contained errors.

After publication of this Article it has been brought to Authors’ attention that Reference 79, which was:

“Latifi-Pupovci, H. Association between autoantibodies against thyroid stimulating hormone receptor and thyroid diseases. *Med. Archiv.* **68**, 79–81 (2014).”

was retracted in 2016. The Authors had missed it because the status of the paper was not up to date in the database they used. This reference was cited in the section 'Knowledgebase utilities and case studies' as follows:

“Grave’s disease is an autoimmune disorder characterized by the production of autoantibodies against TSHR that causes hyperthyroidism^77,78,79^. On the other hand, Hashimoto’s thyroiditis (HT) is an autoimmune disorder that is characterized by the production of autoantibodies against thyroid peroxidase (TPO) that causes hypothyroidism^79,80^.”

This reference has now been removed, and subsequent references have been re-ordered. These changes do not affect the conclusions of the Article.

The original Article has been corrected.

